# Child Sexual Abuse and Suicidal Ideation: The Differential Role of Attachment and Emotional Security in the Family System

**DOI:** 10.3390/ijerph17093163

**Published:** 2020-05-02

**Authors:** David Cantón-Cortés, María Rosario Cortés, José Cantón

**Affiliations:** 1 Department of Developmental and Educational Psychology, Faculty of Psychology, University of Málaga, 29071 Málaga, Spain; 2 Department of Developmental and Educational Psychology, Faculty of Psychology, University of Granada, 18011 Granada, Spain; mcortes@ugr.es (M.R.C.); jcanton@ugr.es (J.C.)

**Keywords:** child sexual abuse, suicidal ideation, attachment, emotional security

## Abstract

The objective of this study was to analyze the effects of attachment style and emotional security in the family system on suicidal ideation in a sample of young adult female victims of child sexual abuse (CSA). The possible effects of CSA characteristics and other types of child abuse on suicidal ideation were controlled for. The sample consisted of 188 female college students who had been victims of sexual abuse before the age of 18, as well as 188 randomly selected participants who had not experienced CSA. The results showed that both attachment and emotional security were associated with suicidal ideation, even when controlling for both the characteristics of abuse and the existence of other abuses. The strong relationships of emotional security and attachment style with suicidal ideation suggest the importance of early intervention with children who have been sexually abused and their families, in an effort to optimize their attachment style, as well as to decrease emotional insecurity to prevent the onset of symptomatology related to suicidal ideation.

## 1. Introduction

Sexual violence refers to a wide range of behaviors, such as attempting or completing intercourse, unwanted sexual contacts, and sexual comments or insinuations made by any individual, regardless of their relationship with the victim [1]. In turn, child sexual abuse (CSA) can be defined as contacts and sexual interactions between a minor and an adult or between minors if there is a 5-year age difference between them or if the child/adolescent perpetrator is in a situation of power or control over the victim, even if there is no age difference [2].

Most of the studies that have examined the aftermath of CSA have extensively documented mental health problems associated with the abuse, one of the most prevalent being suicidal ideation [3,4,5]. Previous research has shown that between 20% and 22% of suicide and suicide attempts among women can be attributed to CSA exposure [6]. However, there is no evidence that there is a consistent group of symptoms that make up a “post-abuse syndrome” and not all victims of child abuse show subsequent significant harm [7]. Recognizing that not all CSA experiences are the same, current research has focused on examining the variables that would explain the differences in victims’ subsequent adjustment [8,9]. Although early research focused on analyzing the role of the characteristics of abuse, more recent research has shown that socio-cognitive factors play a more important role in determining the victim’s psychological state [7]. These factors include variables such as family support received [10], the response obtained after the disclosure of the abuse [11], or self-blame [8]. This study focuses on the role of the family system’s attachment style and emotional security in suicidal ideation.

### 1.1. Attachment

As proposed by Bowlby [12], the attachment theory stems from the idea that interpersonal relationships and social support are an innate need in human beings. Children develop internal working models of significant others, depending on the quality of their early interpersonal experiences (e.g., caregiver response and support). Based on these models, the child creates a series of expectations about the availability of support and how to obtain that support and protection [13]. From a theoretical point of view, these representations influence people’s emotions, expectations, and social behaviors in all their close relationships.

Based on Bowlby’s work, Ainsworth and Wittig [14] identified three attachment styles: secure, avoidant, and anxious-ambivalent. Secure attachment develops when the primary caregiver responds to the child’s discomfort and distress consistently, thus promoting trust in interpersonal relationships. An avoidant attachment style develops when the child’s discomfort is systematically ignored or continually rejected. This pattern leads to the avoidance of seeking attachment figures who provide support and protection and decreases the ability to express emotions appropriately. Finally, an anxious-ambivalent attachment style develops when caregivers respond inconsistently to a child’s anxiousness, leading to a high level of anxiety and difficulty in emotional expression.

Research has shown that the effects of attachment style extend into adulthood and, among other aspects, can influence people’s parenting skills and peer relationships, as well as their romantic relationships [15]. It has been suggested that a secure attachment style is related to healthy regulation of negative affect and may act as a protective factor in the ability to reduce anxiety arising from stressful events. Conversely, an insecure attachment style leads to excessive and unproductive attention to negative emotions or to avoiding feelings of discomfort, leading to inadequate emotion regulation [16].

The effects of attachment style on the psychological adjustment of victims of abuse have been analyzed in recent years [17,18,19]. Nelson and collaborators [19], for example, analyzed the effects of the avoidant and anxious attachment styles on posttraumatic growth in a sample of adult female victims of CSA. The avoidant attachment style and, especially, the anxious attachment style were both related to lower scores in posttraumatic growth.

### 1.2. Emotional Security in the Family System

The Emotional Security Theory (EST) [20] was originally developed to explain how interparental conflict influences children’s development. According to the EST, maintaining a sense of security within the family—including a context of conflict between parents—is vital for the development of children and adolescents. The EST identifies three security patterns for minors in the family system [21]: secure, preoccupied, and disengaged.

The pattern of emotional security refers to the sense of safety, stability, and well-being that arises from family relationships perceived as positive and stable, even in the face of common stressors such as interparental conflict, and is associated with children’s fewer psychological difficulties [22]. For example, the fact that a child witnesses how their parents cope with and resolve their conflicts in a way that maintains family harmony creates trust in the family as a source of security [21].

Conversely, if children are exposed to frightening behaviors within their family, family members are inaccessible, or the family’s response to the child’s anguish or fear is inadequate, the child’s trust in the family system as a source of emotional security will deteriorate. Accordingly, the EST proposes that children actively modify their reality to preserve the feeling of emotional security [20]. Two strategies can be used to modify their family representations: disengagement and preoccupation. The disengagement strategy is the inclination to minimize the importance of the family system and to detach oneself emotionally from it. On the other hand, children who employ preoccupation strategies bias stressful family events, exaggerating their importance to preserve their emotional security [23].

In the short term, not only disengagement but also preoccupation can be adaptive in the context of family conflict and stress. Distancing oneself from the family can function as a method of tolerating discomfort in situations of family instability. On the other hand, the preoccupied child’s hypervigilance of family relationships can provide resources to effectively recognize signs of threat in the family system which, in turn, can stimulate strategies to quickly cope with stress [24].

However, the EST posits that despite the short-term usefulness of the disengagement and preoccupation strategies, the psychological and physical resources used to maintain security reduce the resources available to other processes that are needed for development. Thus, as numerous longitudinal and cross-sectional studies have shown, children using such strategies are at increased risk of developing difficulties such as eating disorders [25], symptoms of hyperactivity [26], adaptation problems in school [27], or addictions [28]. Although numerous studies have tested the EST in the general population, to date, only one work has analyzed the relationship between emotional security/insecurity in the emotional system and mental health of CSA victims [29].

Although the EST shares some of the basic premises of the attachment theory (for example, a security-based system or the concept of internal work models), they also differ significantly. The attachment theory focuses primarily on how children form dyadic relationships with an attachment figure to maintain a sense of security [12]. However, the child’s emotional security in the family system is a significant variable in the context of multiple relationships. In contrast to the attachment theory, the theory of emotional security emphasizes that in addition to the attachment between parents and children, many family characteristics such as family violence and interparental conflict can be a direct obstacle to maintaining security [21].

### 1.3. Objectives

One of the basic assumptions of the theory of emotional security in the family system is that emotional security is relatively independent of the security of the parent-child attachment in its substance, family correlates, and implications for child adjustment [30]. As a result, emotional security in the family system is expected to influence psychological adjustment, even when considering the role of parent-child attachment.

In this sense, Davies and collaborators [30] found that emotional security in the context of interparental conflict and attachment to parents independently influenced the internalizing and externalizing problems of a group of adolescents from the general population. To date, however, no study has simultaneously assessed the role of emotional security and attachment to parents in the psychological adjustment of a sample of victims of CSA.

The objective of this study was to analyze the effect of emotional security in the family system and attachment style on suicidal ideation in a sample of university students who were victims of CSA, controlling for the characteristics of the CSA and the existence of other types of child abuse.

## 2. Materials and Methods

### 2.1. Participants

Of 2328 female first-year students from the University of Granada who were requested to participate in the study, 312 (13.4%) declined. The 2016 students who accepted were between 18 and 24 years old (M = 19.37, SD = 1.67) and completed a survey on the possible experience of CSA and its characteristics. The definition of CSA quoted in the Introduction [2] was the one adopted in the study.

**Figure 1:**
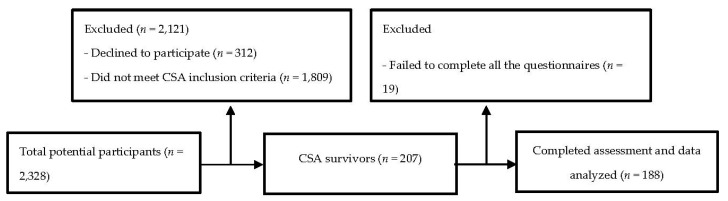
Flow chart for the recruitment of child sexual abuse (CSA) participants.

Of the total participants, 207 (10.26%) reported experiencing some form of sexual abuse involving physical contact before the age of 18. Nineteen of these participants did not complete some of the questionnaires and were removed from the study, so the CSA final sample of the study consisted of 188 victims (current mean age of 19.55 years, SD = 1.71) (Figure 1). A comparison group, made up of the same number of randomly selected participants who had not experienced CSA, was also included in the study.

Of the CSA survivors, 23.9% (n = 45) had suffered abuses consisting of oral sex or penetration, 62.8% (n = 118) had experienced touching, and 13.3% exhibitionism (n = 25). In 53.7% of the cases (n = 101), the CSA consisted of an isolated incident; in 26.6% (n = 50), there had been several incidents (between 2 and 5); and in 19.7% (n = 37), CSA had been continued over time (more than 5 incidents). Ninety eight survivors (52.1%) had suffered abuse from a family member. The average age at which the abuse occurred or began was 9.14 years (SD = 3.37). Finally, of these CSA victims, 17.6% (n = 33) had also suffered emotional or physical abuse or neglect during childhood.

Among the victims, 71.3% came from intact families, 12.2% from families of divorced parents, 3.7% had experienced the death of one or both parents, and 11.2% came from a reconstituted family. In terms of the size of the participants’ family, 9.6% were only children, 51.6% had one sibling, 23% had 2 siblings, 10.2% 3 siblings, and the remaining households (5.6%) were made up of 4 or more siblings.

Regarding non-CSA victims, the average age at which the crisis occurred or began was 11.27 years (SD = 3.42). In terms of other forms of maltreatment during childhood, 15.42% (n = 29) had suffered. 73.9% came from intact families, 11.1% from families of divorced parents, 3.1% had experienced the death of at least one parent, and 11.7% came from a reconstituted family. With regard to the size of the participants’ family, 8% were only children, 56.4% had one sibling, 28.2% had 2 siblings, 5.9% 3 siblings, and the remaining households (1.6%) were made up of 4 or more siblings.

### 2.2. Instruments

Child Sexual Abuse Questionnaire. This is a survey-like questionnaire that collects sociodemographic and CSA-related information confidentially. Participants report their sex, age, family structure, and number of siblings and also answer a series of questions about their possible experiences of CSA. The questionnaire provides participants with the above-mentioned definition of CSA to identify the abusive experience. Next, participants are asked to indicate the nature of the sexual activity (ranging from those that did not involve physical contact to those consisting of touching erogenous zones and finally, those involving penetration and/or oral sex), continuity of abuse (continuous abuse, several incidents, or an isolated incident), and the age at which the sexual abuse occurred or began in the case of continued SCA. The above information was used for participant inclusion/exclusion criteria.

This questionnaire also collects information about other types of abuse and neglect during childhood. Five questions are asked about emotional abuse (e.g., How often did a parent or caregiver act in a way that made you fear physical harm?), physical abuse (e.g., How often did a parent or caregiver slap or beat you?), and negligence (e.g., How often did a parent or caregiver ignore your need for affection?). These questions are rated on a five-point Likert scale ranging from 1 (never) to 5 (very often). Participants were identified as victims of emotional abuse, physical abuse, or neglect if they responded often or very often to at least one question.

Security in the Family System Scale (SIFS) [21]. Items measuring this variable are rated on a 4-point Likert scale, ranging from 1 (strongly disagree) to 4 (strongly agree). The scale evaluates three patterns of security/emotional insecurity. The Security scale assesses the victim’s trust in the family as a reliable source of support and protection (7 items; e.g., I believe that family members will be around to help me in the future). The Preoccupation scale assesses concerns about the future well-being of the family and oneself as a member of that family (8 items; e.g., Sometimes, I feel that something very bad is going to happen in my family). Finally, the Disengagement scale measures the victim’s efforts to disassociate and minimize the importance of the family (7 items; e.g., When something bad happens in my family, I wish I could live with a different family). The Cronbach alpha coefficients obtained in this study were 0.87 (Security), 0.85 (Preoccupation), and 0.83 (Disengagement).

Attachment Style Measure (ASM) [31]. The ASM is a 13-item self-report scale, based on the Adult Attachment Styles (AAS) [32]. The AAS was developed from the descriptions of secure, avoidant, and anxious-ambivalent attachment styles. Using the AAS, participants rate themselves according to one of the three attachment vignettes, each one representing one of the mutually exclusive attachment categories. The objective of the ASM is to establish a more accurate measurement of the attachment style by assessing the three categories of the AAS with 13 items, such that each item corresponds to one of the three attachment vignettes of the AAS. The responses are rated on a 7-point Likert-type scale ranging from 1 (strongly disagree) to 7 (strongly agree). Thus, the scale evaluates three types of adult attachment: Secure (5 items; e.g., I know others will be there when I need them), Avoidant (4 items; e.g., I am somewhat uncomfortable being close to others), and Anxious (4 items; e.g., I find that others are reluctant to get as close as I would like). Sperling, Foelsch, and Grace [33] found that the ASM was the best instrument for continuously evaluating attachment. Cronbach alpha of 0.71 for the Secure style, 0.67 for the Anxious style, and 0.77 for the Avoidant style were found in this study.

Scale for Suicide Ideation (SSI) [34]. This evaluates and quantifies the intensity of suicidal ideation through various dimensions of self-destructive thoughts or desires. It presents 19 items, each with 3 response alternatives in an intensity gradient ranging from 0 to 2 (e.g., Duration of suicidal ideation/wish: 0 = Brief, fleeting periods; 1 = Longer periods; 2 = Continuous —chronic— or almost continuous). These items assess the existence of thoughts about suicide and their characteristics, as well as the participant’s attitude towards them; the intensity of the desire to die, the desire to make a real suicide attempt, and the details of the plans if they exist; internal deterrents to an active attempt; and subjective feelings of control and/or “courage” regarding a proposed attempt. To learn about the differences in suicidal ideation at the time of the abuse compared with the present, participants were asked to respond to the scale in duplicate, thinking first of all about the time of crisis and then in the present. Cronbach alpha coefficients obtained in this study were 0.77 for suicide ideation at the time of crisis and 0.66 at present.

### 2.3. Procedure

The study was conducted in accordance with the Declaration of Helsinki and approval of the Granada University Ethics Committee was obtained for all protocols and materials used in this study (UGR-022005). The students were informed that participation was voluntary and confidential and their informed written consent was requested. In a classroom, participants completed the CSA Questionnaire, aimed at identifying CSA victims, the characteristics of the abuse, and the possible existence of other maltreatment. They also completed the Security in the Family System Scale (SIFS to assess the security, preoccupation, and disengagement of the family system), Attachment Style Measure (ASM; for secure, avoidant, and anxious attachment), and Scale for Suicide Ideation (SSI; for the evaluation of suicidal ideation at the time of crisis and in the present).

Participants who had been abused by different aggressors at different times were asked to respond to questionnaires regarding the experience that was the most traumatic for them. Participants were told that they could stop completing the questionnaires at any time if they felt uncomfortable during the study. To maintain the confidentiality of the CSA victims, students who had not been abused completed the questionnaires concerning some negative life experience. Psychological counseling was offered by members of the research team to all participants after the completion of the study.

## 3. Results

The effects of CSA were analyzed by comparing the scores in suicidal ideation at the time of crisis and at present between the CSA victim group and the comparison group, made up of the same number of randomly selected participants who had not experienced CSA (Table 1). Non-victims were asked to answer thinking of the most negative experience that they had suffered during childhood or adolescence. The mean difference was statistically significant only in the case of the suicidal ideation at the time of crisis, with CSA victims presenting higher scores than non-victims (Mann–Whitney *U* = 12,191.50, *p* < 0.001).

Table 2 presents the descriptive statistics of the attachment, emotional security, and suicidal ideation scales among CSA victims. To examine the role of attachment and emotional security of the family system in suicidal ideation, Pearson’s correlations between the characteristics of the abuse (age at onset, type of abuse, continuity of abuse), the existence of other maltreatment, attachment styles (secure, avoidant, and anxious attachment), and emotional security (security, preoccupation, and disengagement) and suicidal ideation were carried out. Pearson’s correlations were also calculated between attachment styles, emotional security, and suicidal ideation among non CSA victims. The resulting correlation matrixes are presented in Table 3 and Table 4.

Regarding CSA survivors, at the time of crisis, significant correlations were found between suicidal ideation and the characteristics of the abuse, the existence of another maltreatment, and the three variables of attachment and emotional security in the family system. Concerning present suicidal ideation, no correlation was found with the characteristics of the abuse, but a relationship was observed with the existence of another maltreatment and the three variables of attachment and emotional security. Significant correlations were also found between emotional security and the attachment variables. With regard to non-victims of CSA, correlations of attachment and emotional security with suicidal ideation at crisis and present were lower than those found in the case of CSA victims.

To compare the relative effects of attachment and emotional security in the family system on suicidal ideation at the time of crisis and at present, as well as the proportion of variance explained by each variable, a linear multiple regression analysis was carried out for each of the two suicidal ideation variables. The characteristics of the abuse (age at onset, type of abuse, and continuity of abuse) and the existence of other maltreatment were also introduced in the analysis to control for their effects on suicidal ideation.

The regression model for suicidal ideation at the time of crisis (Table 5) was statistically significant, *F* (1, 182) = 11.79, *p* < 0.001, with *R^2^* = 0.24. The variables related to suicidal ideation were age at onset, β = 0.20, *p* < 0.01, continuity of abuse (β = 0.16, *p* < 0.05), and other maltreatment (β = 0.14, *p* < 0.05), such that higher scores were obtained in suicidal ideation when the abuse began later, had been prolonged over time, and some other type of abuse had been suffered. A direct relationship was also found with anxious attachment (β = 0.20, *p* < 0.01) and disengagement (β = 0.21, *p* < 0.01).

Lastly, the regression model for suicidal ideation at present (Table 6) was also statistically significant, *F* (1, 184) = 14.17, *p* < 0.001, with *R^2^* = 0.19. In this case, suicidal ideation was again associated with age at onset (β = 0.15, *p* < 0.05), such that higher scores were obtained in suicidal ideation when the abuse began later. A direct relationship of suicidal ideation was also found with anxious attachment (β = 0.17, *p* < 0.01), as well as an inverse relationship with emotional security (β = −0.33, *p* < 0.001).

## 4. Discussion

Reducing the incidence of CSA is obviously important, but it is equally important to develop intervention strategies focused on the factors that modulate the mental health of CSA victims [35]. This study advances in this direction, analyzing the simultaneous effect of emotional security in the family system and the attachment style with suicidal ideation at the time of crisis and at present, in a sample of college student victims of CSA.

This research corroborates the results of previous studies that have found a link between CSA and suicidal ideation during childhood and adolescence [6]. Thus, our results show that CSA victims have higher rates of suicidal ideation at the time of crisis than another sample from the general population. However, this investigation did not find any difference in current suicidal ideation in a sample of victims and non-victims of CSA. This result may be due to the characteristics of the sample of this study, a university population with higher functioning than the general population (e.g., at a cognitive level) [36].

In terms of the simultaneous role of emotional security and attachment style, the regression models vary considerably depending on the suicidal ideation variable analyzed. Suicidal ideation at the time of crisis was related to the characteristics of the age at the onset of CSA and the continuity of the abuse, as well as to the existence of other maltreatment. Previous studies have shown how polyvictimization, consisting of suffering various types of child abuse, is related to worse psychological adjustment [37]. Concerning the characteristics of the abuse, whereas some authors have found a relationship between these variables and the victims’ psychological adjustment [38], others have not found this relationship [39].

A relationship with anxious attachment was also found, whereas the relationships with secure and avoidant attachment proved to be nonsignificant. These results are consistent with the studies of Lutz-Zois and collaborators [40] or Unger [41], who found that an anxious attachment style was linked to an increased risk of negative psychological consequences in a sample of female CSA victims. However, as our results did not reveal any relationship of secure and avoidant attachment styles with suicidal ideation at the time of crisis, they contradict studies such as that of Unger [41] or Nelson and collaborators [19], who found that avoidant attachment mediated the relationship of CSA with psychological stress and posttraumatic growth, respectively.

Finally, concerning emotional security, a link with suicidal ideation at the time of crisis was only found regarding disengagement. This result coincides with that of a previous study that found no security effect, but it did find an effect of disengagement on the psychological adjustment of CSA victims [29]. However, unlike this last study, no relationship of preoccupation was found with victims’ psychological adjustment. However, in the aforementioned study, the adjustment variable used was not suicidal ideation, but psychological distress, evaluated with the clinical scale SCL-90-R. Thus, these differences with other previous studies regarding the role of attachment and emotional security in victims’ psychological adjustment may be due to the simultaneous evaluation of these variables in this study.

Concerning present suicidal ideation, the percentage of explained variance was lower than in the case of suicidal ideation at the time of the abuse. The difference in the explanatory value of the variables in the study compared to their value in the case of suicidal ideation during the crisis may be due to the low levels of suicidal ideation currently found in our sample. Regarding the characteristics of the abuse, a link of present suicidal ideation was found only with age at onset, with the remaining variables, and the existence of other maltreatment being nonsignificant. Regarding the attachment style, again, only anxious attachment was related to suicidal ideation. Finally, with regard to emotional security in the family system, security rather than disengagement was related to suicidal ideation.

Considering that one of the basic assumptions of the emotional security theory is that security is relatively independent of the security of the parent-child attachment, it has been hypothesized that emotional security in the family system would influence children’s psychological adjustment, even taking into account the role of parent-child attachment. The results of this study confirm this hypothesis, having simultaneously found variables from both theories related to suicidal ideation at the time of crisis and at present. In this sense, our results are consistent with those of Davies and collaborators [30], who found a simultaneous effect of attachment style and emotional security on internalizing and externalizing symptomatology in a sample of adolescents of the general population.

Although this research makes important contributions to the literature on the psychological adjustment of victims of CSA, it also has several limitations that should be mentioned. First, a cross-sectional design was used, with a single source of information to assess emotional insecurity, attachment style, and suicidal ideation simultaneously, with the consequent risk of confusing mediating variables with the consequences of CSA. In the absence of a prospective design, it is not possible to determine which of these variables is the cause and which is the effect. Also, information about current or past mental disorders was not included in the regression models.

Another limitation of the study arises from its retrospective nature, which is often considered to be unreliable. However, retrospective research based on self-reports could be the best approach to some forms of child abuse, such as CSA, as this often goes unnoticed by informants other than the victim themselves [42]. More than 90% of CSA victims never disclose it publicly, so retrospective studies are the only means to obtain information about these cases [7]. Moreover, some researchers have argued that retrospective designs with young adults may be more advantageous for reducing biases linked to this type of research by preventing memory distortions that older adults might have [43], which is why this study limited the maximum age of participants to 24 years.

Moreover, Chronbach’s alpha for the ASM anxious style and SSI suicidal ideation at present were slightly below the recommended values. Specifically, the internal consistency found for the present suicidal ideation could explain why an effect of CSA was not found regarding this scale and also the lower regression coefficients found in this analysis.

One last limitation involves the applicability of the findings to clinical practice. As the participants were university students, the sample in this study could be higher-functioning than non-student young adults (for example, concerning their cognitive abilities) [36], which hinders the applicability of the results obtained to other populations. However, as only a small percentage of CSA victims disclose the abuse, there is still a need to investigate with non-clinical samples [7]. Also, the sample from our study was made up of only female victims of sexual abuse. This investigation focused on female CSA survivors because, as previous research has consistently shown [44], this population is at significantly higher risk for CSA than males. However, the results should be replicated in samples of male victims of CSA. Moreover, other negative experiences could increase the extent of psychological impairment. Experiences such as poly-victimization or family dysfunction during childhood could also have an effect on the psychosocial adjustment of CSA survivors and should be considered in future research [45,46].

## 5. Conclusions

Despite the above limitations, this study contributes to clarifying the role of attachment and emotional security in the family sphere in the mental health of adults with a history of sexual abuse. These findings are very relevant as they can help understand the pathways linking CSA to suicidal ideation among young adults. The optimization of attachment style, as well as a decrease in emotional insecurity, should be essential goals of therapeutic approaches in the treatment of symptomatology related to suicidal ideation.

## Figures and Tables

**Table 1:** Differences in suicidal ideation at crisis and at present among CSA survivors and non-victims (*n* = 376).

	Group	*N*	*Mean*	*SD*	*Mann-Whitney U*
Suicidal Ideation at Crisis	Non-victims	188	2.3	2.71	12,191.50 ***
	Survivors	188	3.9	3.15	
Present Suicidal Ideation	Non-victims	188	0.29	0.86	16,614.50 (NS)
	Survivors	188	0.47	1.27	

*** p < 0.001. NS: non-significant.

**Table 2:** Descriptive statistics of attachment style, emotional security in the family system, and suicidal ideation among CSA survivors (*n* = 188).

Variable	M	SD	Min.	Max.	Skewness	Kurtosis
Secure attachment	11.84	2.07	6	17	−0.05	0.09
Avoidant attachment	11.43	2.63	5	19	0.24	0.07
Anxious attachment	11.18	2.65	6	19	0.27	−0.23
Security in the Family System (SIFS) Security	22.81	4.37	7	32	−1.19	1.44
SIFS Preoccupation	17.27	5.64	8	31	0.25	−0.7
SIFS Disengagement	12.47	4.52	3	28	0.76	0.09
Suicidal Ideation at Crisis	3.91	3.15	0	10	0.38	−1.13
Present Suicidal Ideation	0.47	1.27	0	10	4.37	23.7

**Table 3:** Pearson correlations of all of the variables examined among non-victims (*n* = 188)**.**

Variable	1	2	3	4	5	6	7
1. Secure Attachment							
2. Avoidant Attachment	−0.45 ***						
3. Anxious Attachment	−0.07	0.18 *					
4. SIFS Security	0.18 *	−0.22 **	−0.18 *				
5. SIFS Preoccupation	−0.12	0.27 ***	0.28 ***	−0.37 ***			
6. SIFS Disengagement	−0.09	0.23 **	0.25 ***	−0.75 ***	0.52 ***		
7. S. Ideation at crisis	−0.16 *	0.22 **	0.25 **	−0.27 ***	0.25 **	0.31 ***	
8. Present S. Ideation	−0.08	0.17 *	0.14	−0.1	0.19 *	0.18 *	0.32 ***

* p < 0.05. ** *p* < 0.01. *** *p* < 0.001.

**Table 4:** Pearson correlations of all of the variables examined among CSA survivors (*n* = 188)**.**

Variable	1	2	3	4	5	6	7	8	9	10	11
1. Age of onset											
2. Type of abuse	−0.08										
3. Continuity of abuse	−0.22 **	0.22 ***									
4. Other maltreatment	0.1	0.13	0.13								
5. Secure Attachment	0.06	0.05	−0.14 *	−0.13							
6. Avoidant Attachment	0.05	−0.02	0.14 *	0.20 **	−0.60 ***						
7. Anxious Attachment	−0.09	0.07	0.11	0.20 **	−0.19 ***	0.17 *					
8. SIFS Security	0.05	−0.08	−.18 *	−0.48 ***	0.39 ***	−0.32 ***	−0.27 ***				
9. SIFS Preoccupation	0.01	0.05	0.07	0.29 ***	−0.25 ***	0.31 ***	0.37 ***	−0.58 ***			
10. SIFS Disengagement	−0.01	0.05	0.10	0.38 ***	−0.38 ***	0.33 ***	0.33 ***	−0.77 ***	0.67 ***		
11. S. Ideation at crisis	0.16 *	0.10	0.18 *	0.31 ***	−0.24 ***	0.20 **	0.30 ***	−0.35 ***	0.33 ***	0.35 ***	
12. Present S. Ideation	0.11	0.05	0.05	0.27 ***	−0.26 ***	0.15 *	0.25 ***	−0.37 ***	0.23 ***	0.30 ***	0.34 ***

* p < 0.05. ** *p* < 0.01. *** *p* < 0.001.

**Table 5:** Regression analysis of suicidal ideation at the time crisis as a function of the characteristics of the abuse, the existence of other maltreatment, attachment, and security in the family system (*n* = 188).

Variable	*R^2^*	*F*	β	*t*
	0.24	11.79 ***		
Age at onset			0.2	3.06 **
Type of abuse			0.04	0.68
Continuity of abuse			0.16	2.38 *
Other maltreatment			0.14	2.03 *
Secure attachment			−0.11	−1.59
Avoidant attachment			0.04	0.59
Anxious attachment			0.2	2.98 **
SIFS Security			−0.12	−1.08
SIFS Preoccupation			0.11	1.29
SIFS Disengagement			0.21	2.93 **

* *p* < 0.05. ** *p* < 0.01. *** *p* < 0.001.

**Table 6:** Regression analysis of present suicidal ideation as a function of the characteristics of the abuse, existence of other maltreatment, attachment, and security in the family system (*n* = 188).

Variable	*R^2^*	*F*	β	*t*
	0.19	14.17 ***		
Age at onset			0.15	2.27 *
Type of abuse			0.02	0.39
Continuity of abuse			0.01	0.01
Other maltreatment			0.07	0.99
Secure attachment			−0.12	−1.71
Avoidant attachment			0.01	0.07
Anxious attachment			0.17	2.52 **
SIFS Security			−0.33	−4.85 ***
SIFS Preoccupation			−0.05	−0.6
SIFS Disengagement			−0.03	−0.34

* *p* < 0.05. ** *p* < 0.01. *** *p* < 0.001.
